# Supplementary figures and images for: Immunopeptidomic MHC-I profiling and immunogenicity testing identifies Tcj2 as a new Chagas disease mRNA vaccine candidate
Source: PLoS Pathog. 2024 Dec 18;20(12):e1012764. doi: 10.1371/journal.ppat.1012764 (PMC11654963; doi:10.1371/journal.ppat.1012764)

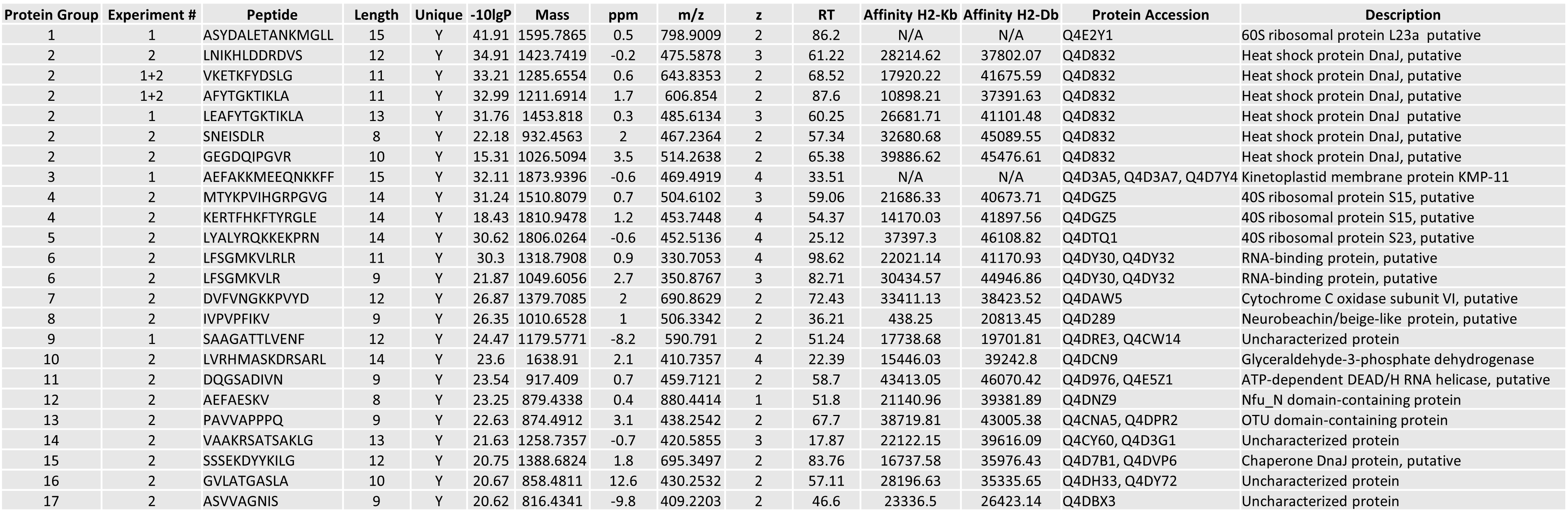

Supplement: S1 Table — Properties of each peptide calculated by mass spectrometry are listed. Affinity (in nM) to H2-Kb and H2-Db was calculated using NetMHCpan 4.1 prediction tool. (TIF) [file ppat.1012764.s001.tif]

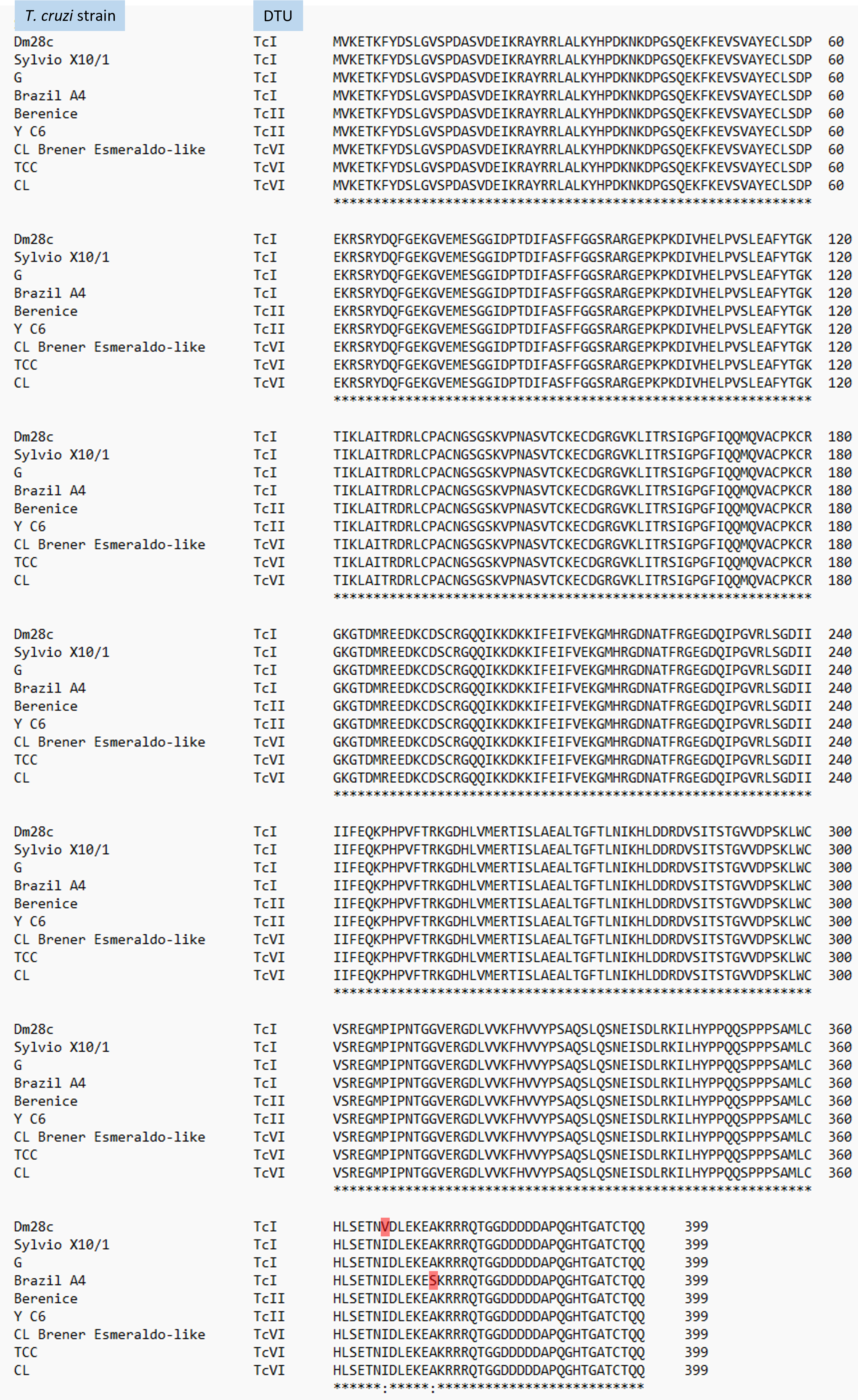

Supplement: S1 Fig — Multiple sequence alignment of DnaJ (Tcj2) protein sequence of different T. cruzi strains, obtained from TriTripDB.org (accessed on May 23rd, 2023). The two mutations in amino acid residues are highlighted in red. (TIF) [file ppat.1012764.s002.tif]

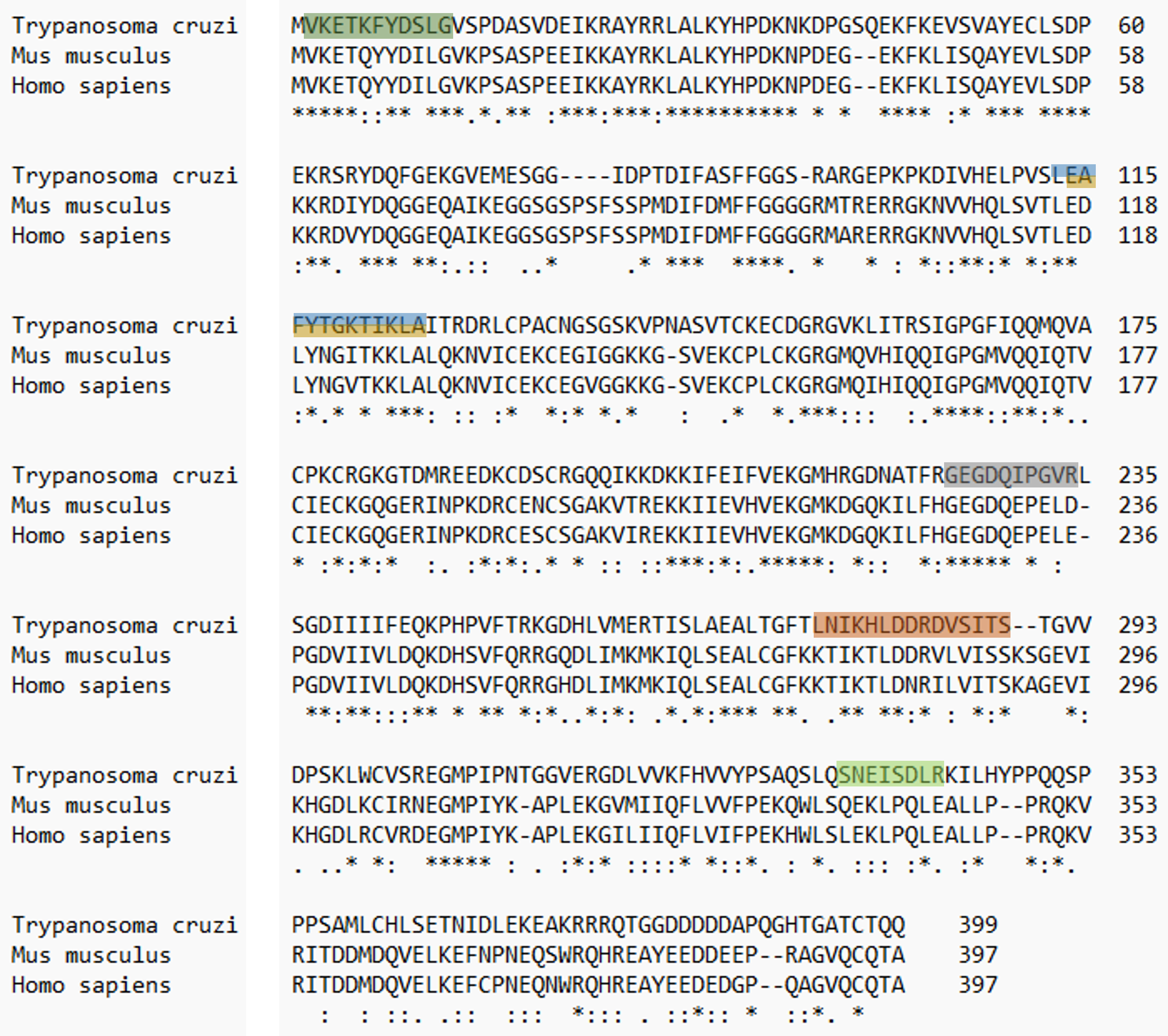

Supplement: S2 Fig — The colored sequences represent the six peptides that were found by immunopeptidomics. Sequence identity is 42% between T. cruzi and M. musculus (dnaJ homolog subfamily A member 4 isoform 2). Sequence identity is 43% between T. cruzi and H. sapiens (dnaJ homolog subfamily A member 4 isoform 2). (TIF) [file ppat.1012764.s003.tif]

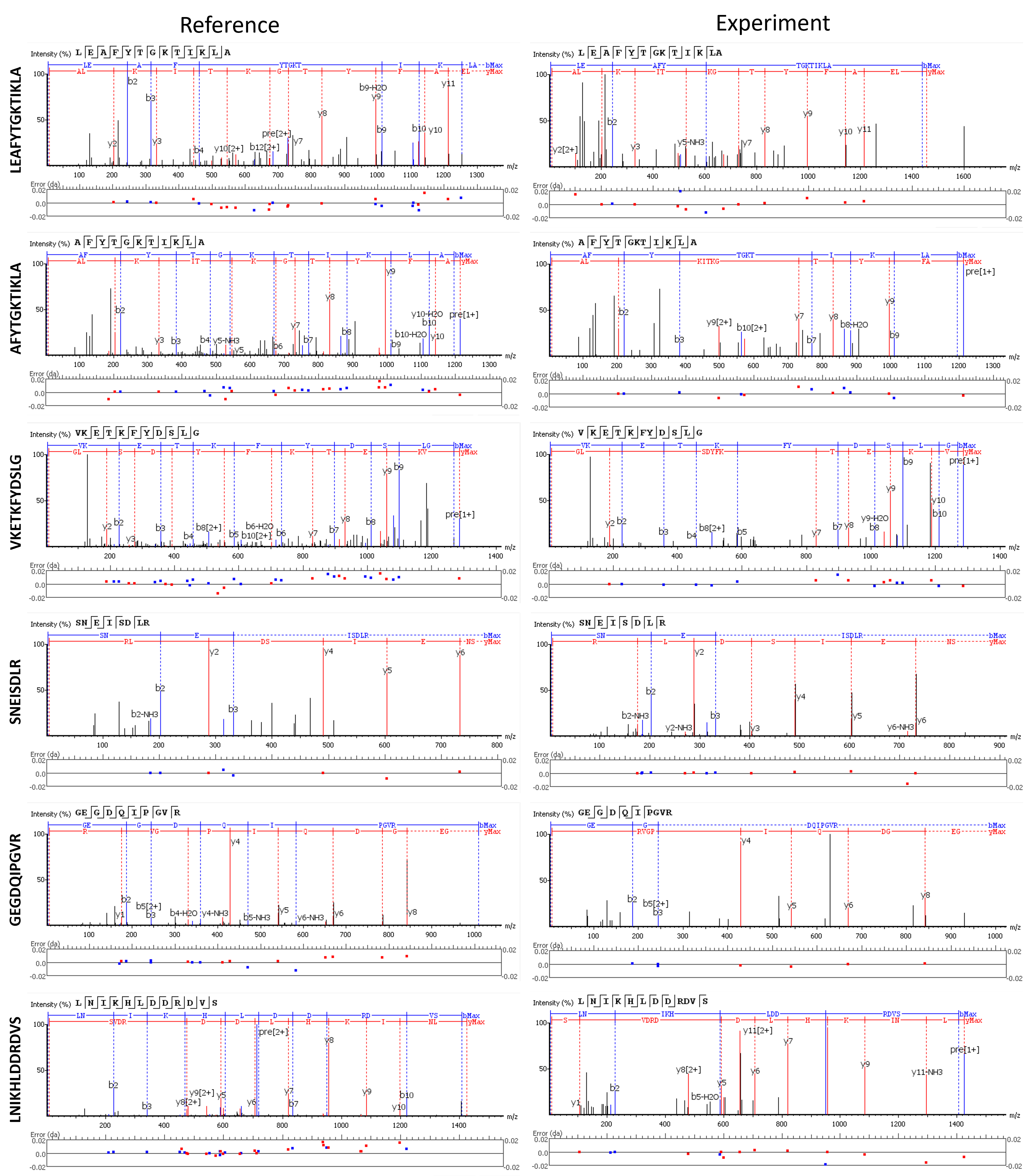

Supplement: S3 Fig — Spectra from Tcj2 reference synthetic peptides were compared to spectra from experimental peptides, and similarities confirmed the identity of these peptides. Corresponding -10LogP values for the experimental peptides were deemed to be significant as determined by the PEAKS algorithm to be 21.4, 30.1, 28.3, 20.7, 17.3, 30.2 from top to bottom. The measured mass error was less than 4 ppm for all peptides. (TIF) [file ppat.1012764.s004.tif]

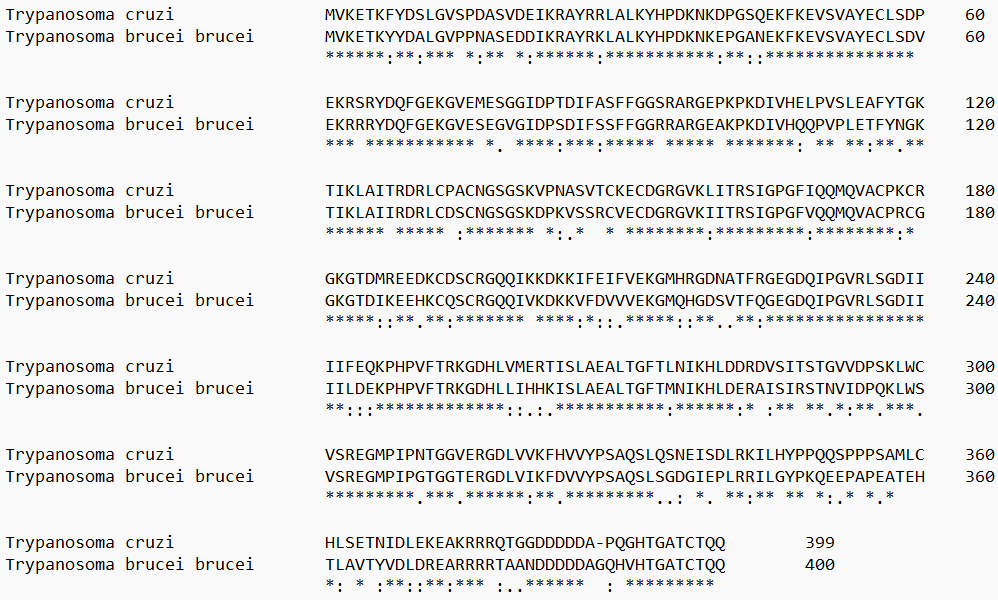

Supplement: S4 Fig — Protein sequence obtained from TriTripDB.org (accessed on July 3rd, 2023). (TIF) [file ppat.1012764.s005.tif]

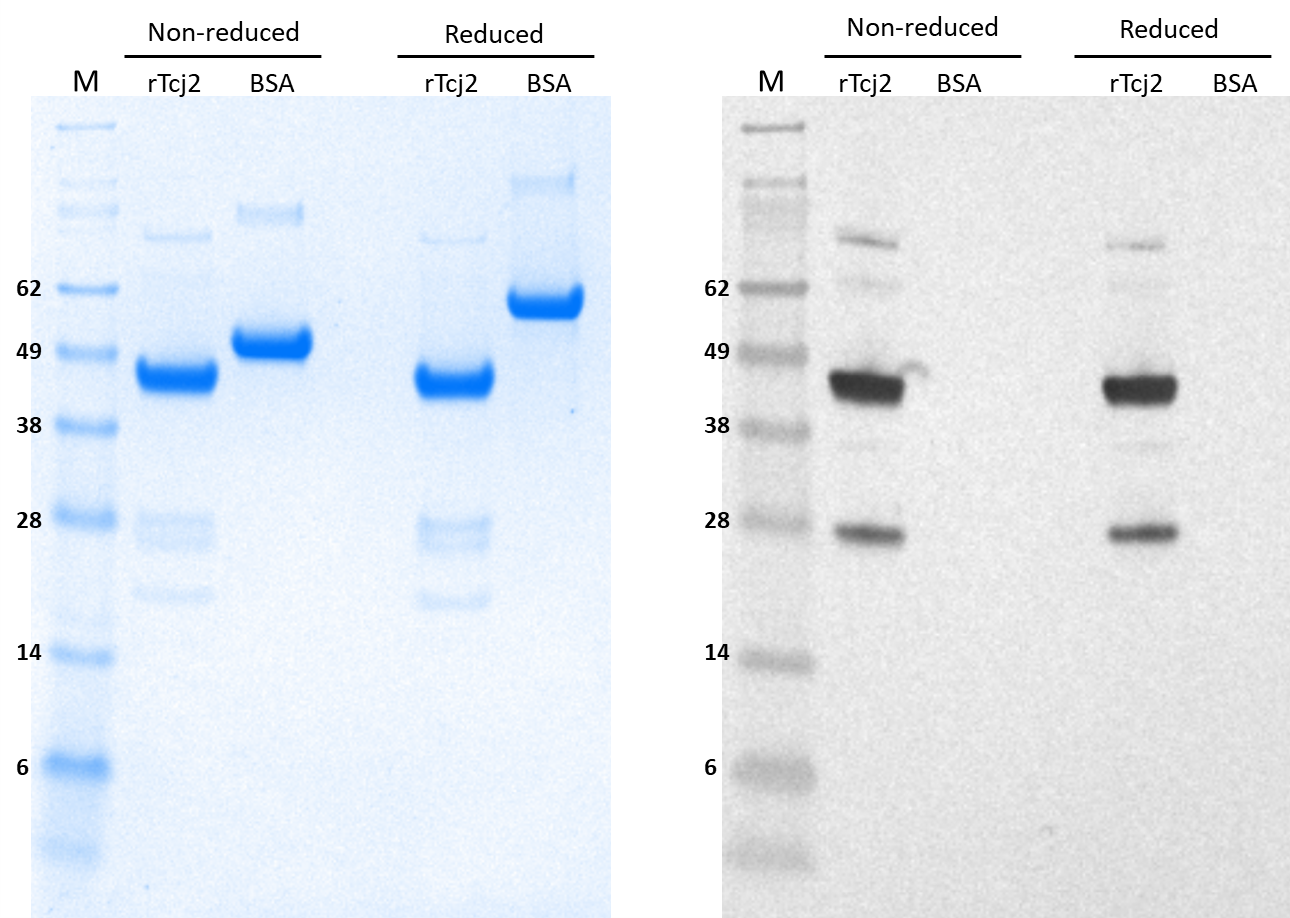

Supplement: S5 Fig — Coomassie stained SDS-PAGE gel and western blot detecting HIS-tag showed a main band at the expected size of 45 kDa. Three bands smaller than the main band were observed, of which one band contained the HIS-tag. Densitometry analysis estimated the purity of the main band to be 76%. (TIF) [file ppat.1012764.s006.tif]

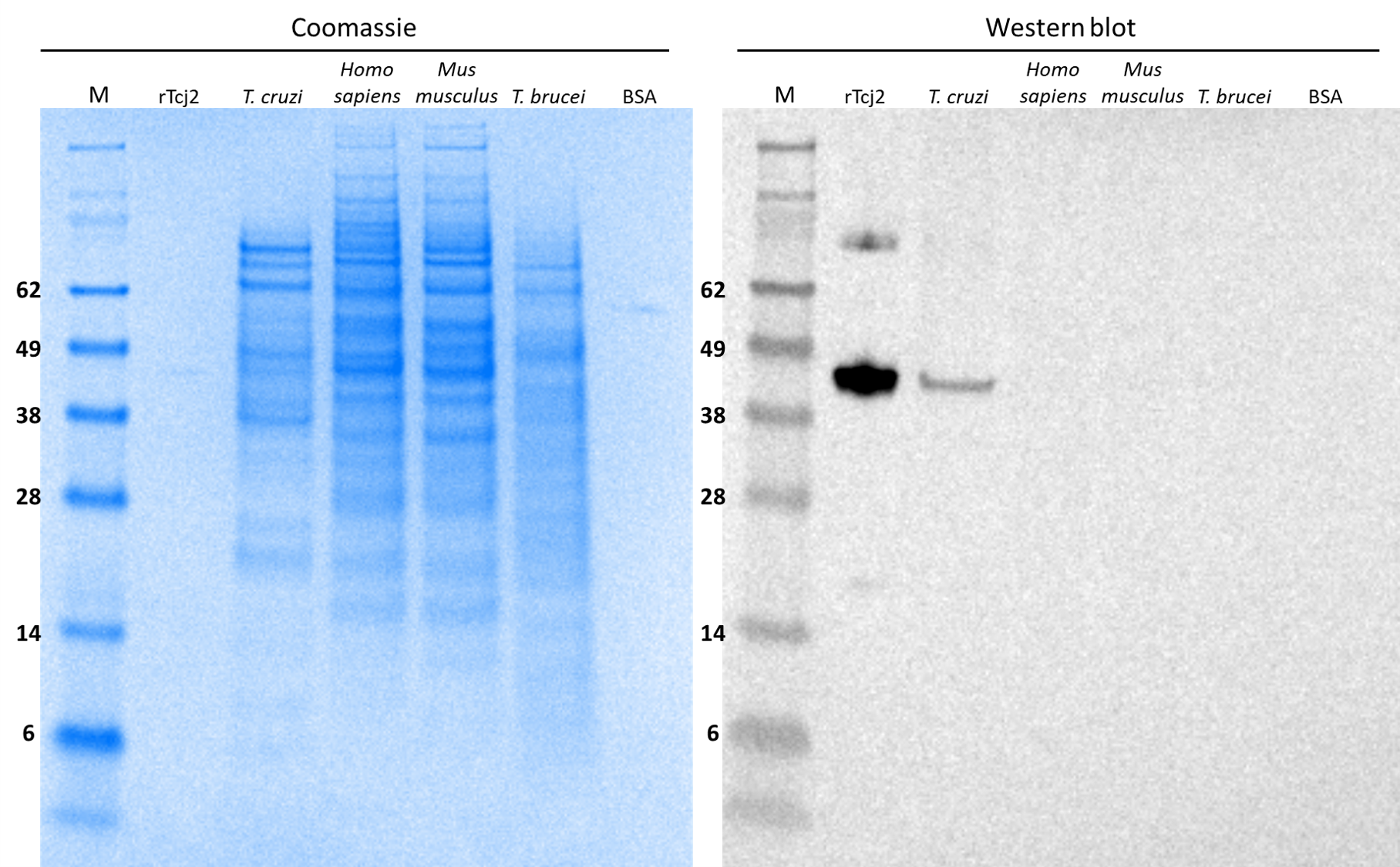

Supplement: S6 Fig — 20 ng rTcj2 protein, as well as 3 μg of lysates from T. cruzi Tulahuen, H. sapiens (HEK293T), M. musculus (MC57G) and T. brucei brucei, were ran on reduced SDS-PAGE gels and either stained with Coomassie Blue or subjected to western blotting followed by incubation with Tcj2 antisera from Tcj2 LNP-vaccinated mice. 20 ng Bovine Serum Albumin (BSA) protein was added as negative control. (TIF) [file ppat.1012764.s007.tif]

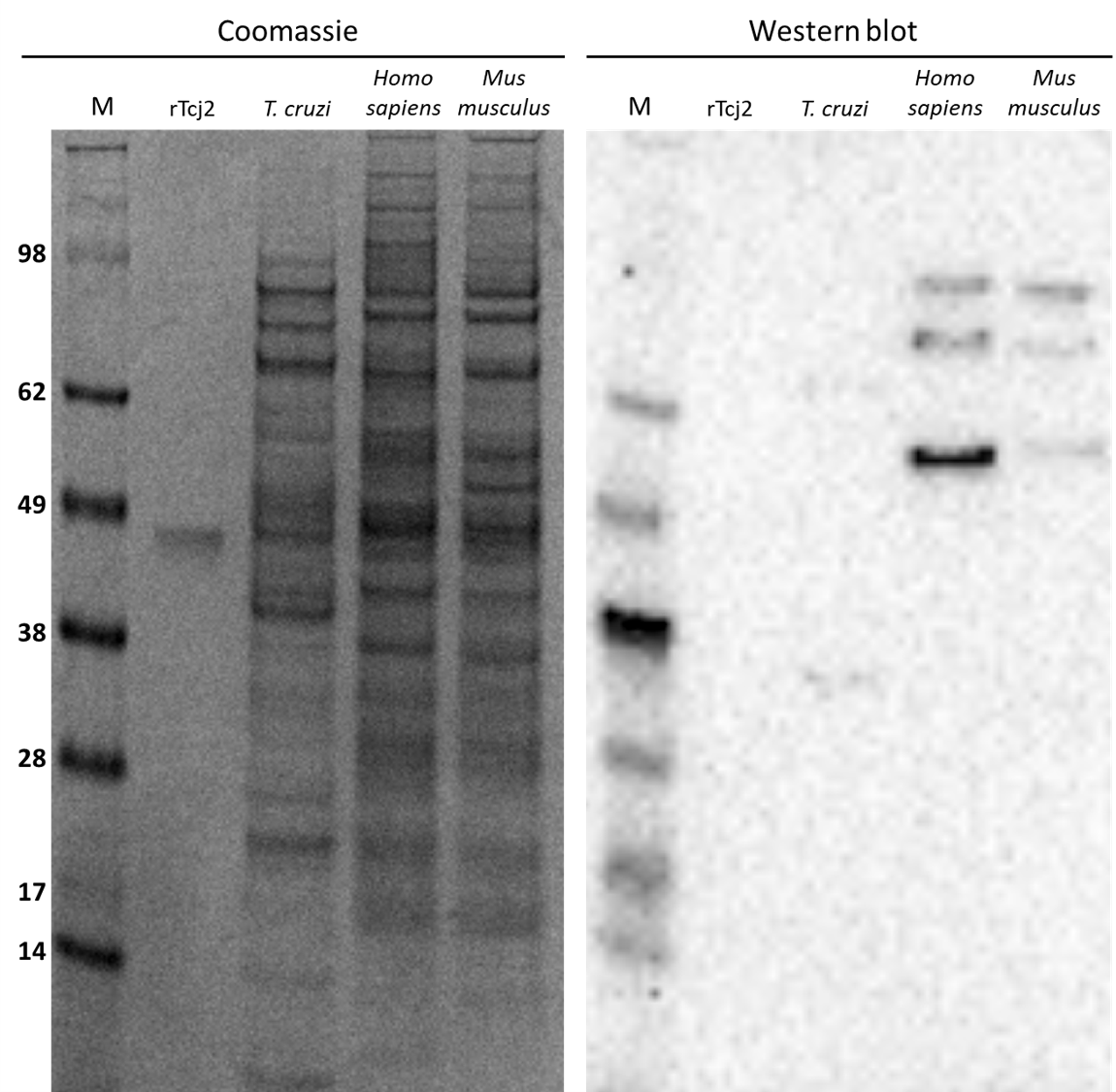

Supplement: S7 Fig — 100 ng rTcj2 protein, as well as 3 μg of lysates from T. cruzi Tulahuen, H. sapiens (HEK293T) and M. musculus (MC57G) were ran on reduced SDS-PAGE gels and either stained with Coomassie Blue or subjected to western blotting followed by incubation with anti-human DNAJA4 polyclonal antibody. (TIF) [file ppat.1012764.s008.tif]

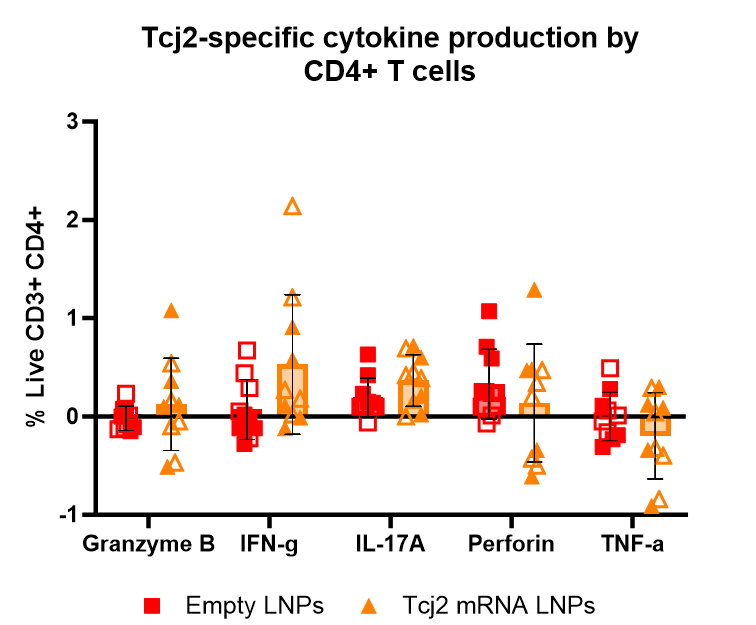

Supplement: S8 Fig — No significant changes in cytokine production were observed by CD4+ T cells after restimulation, but an observable trend in increase in IFN-γ was observed. Data values from non-stimulated cells were subtracted from rTcj2 protein stimulated cells to obtain antigen-specific cytokine production. Mean and standard deviation are shown. Filled symbol shapes represent immunogenicity study #1, while open symbol shapes represent repeat immunogenicity study #2. (TIF) [file ppat.1012764.s009.tif]

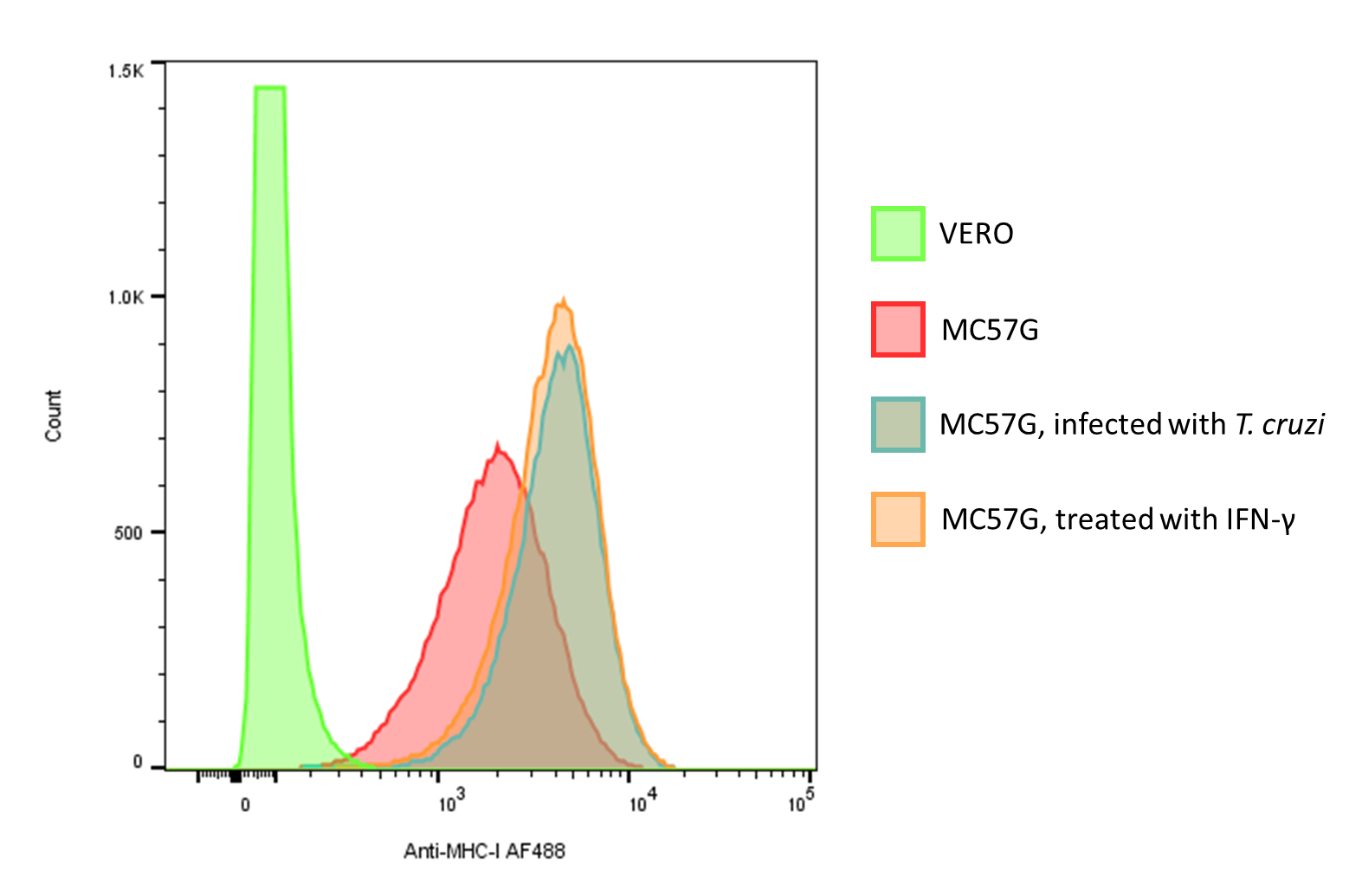

Supplement: S9 Fig — Cells were infected with T. cruzi for 48 hours followed by flow cytometric staining. As a positive control for upregulated MHC-I expression, cells were incubated for 24 hours with recombinant mouse IFN-γ. VERO cells that do not have mouse MHC-I were used as a negative control. (TIF) [file ppat.1012764.s010.tif]

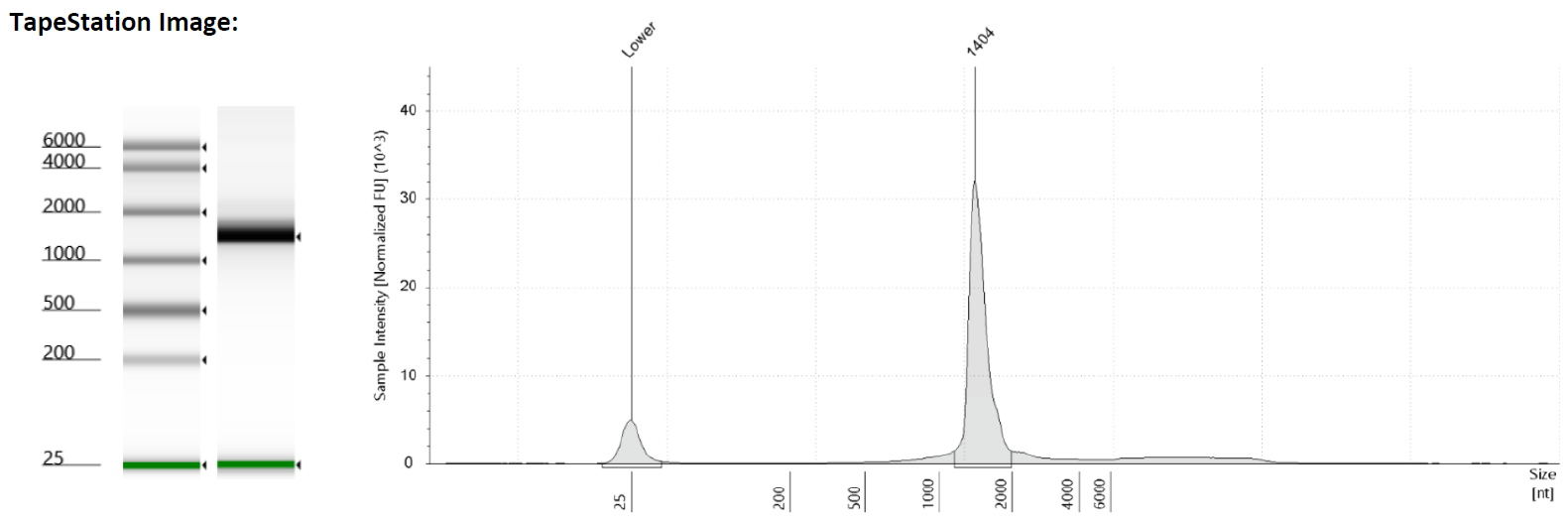

Supplement: S10 Fig — Tapestation automated electrophoresis was performed to analyze the size and integrity of the Tcj2 mRNA construct. A strong single band was observed, calculated to be 1404 nucleotides, which was the expected size. The second band at 25 nucleotides represents a positive control that is run within the sample. (TIF) [file ppat.1012764.s011.tif]

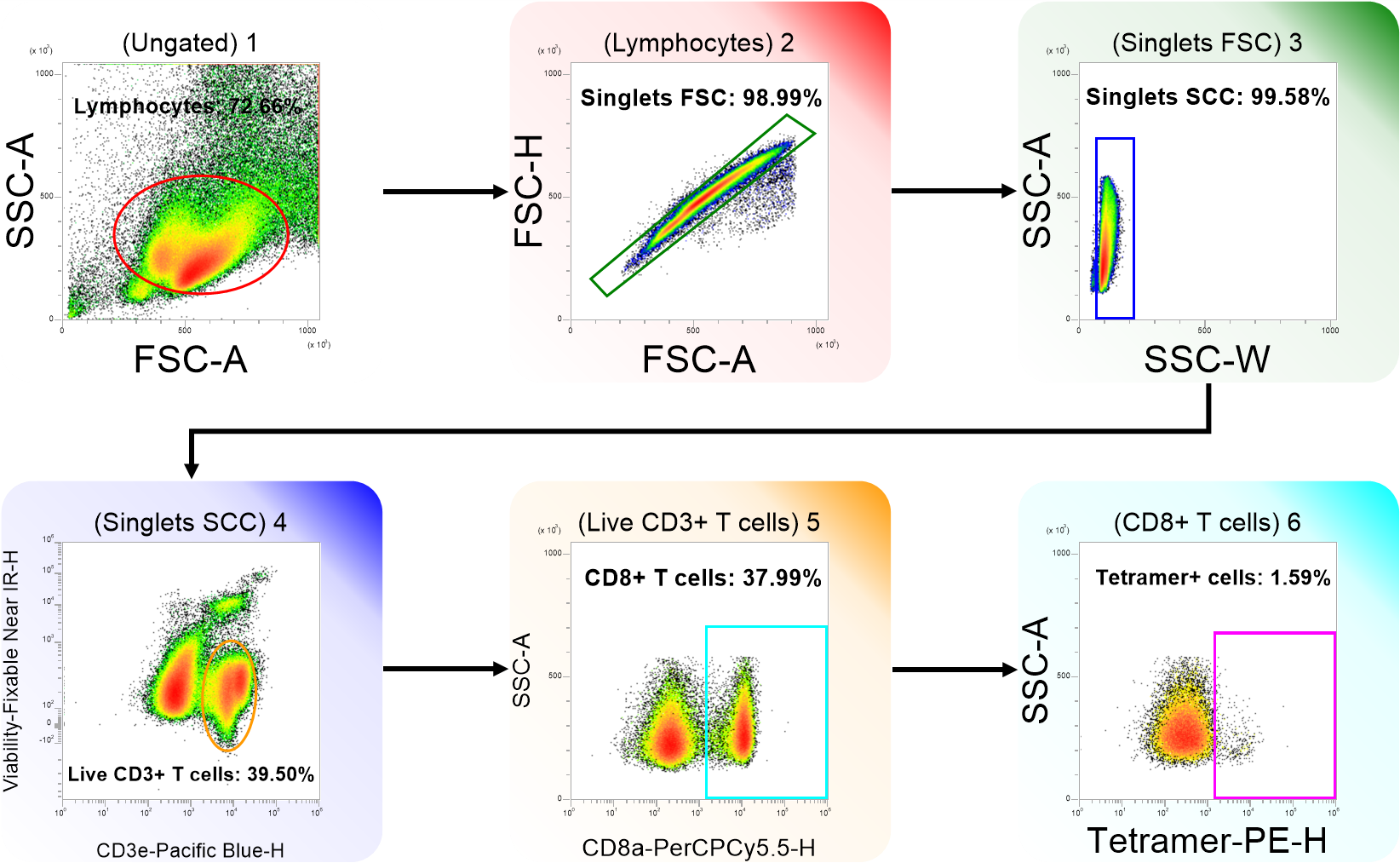

Supplement: S11 Fig — After lymphocytes and singlets were selected, live CD3+ T cells were gated, followed by CD8+ T cells, followed by gating on SIINFEKL tetramer PE positive cells. (TIF) [file ppat.1012764.s012.tif]

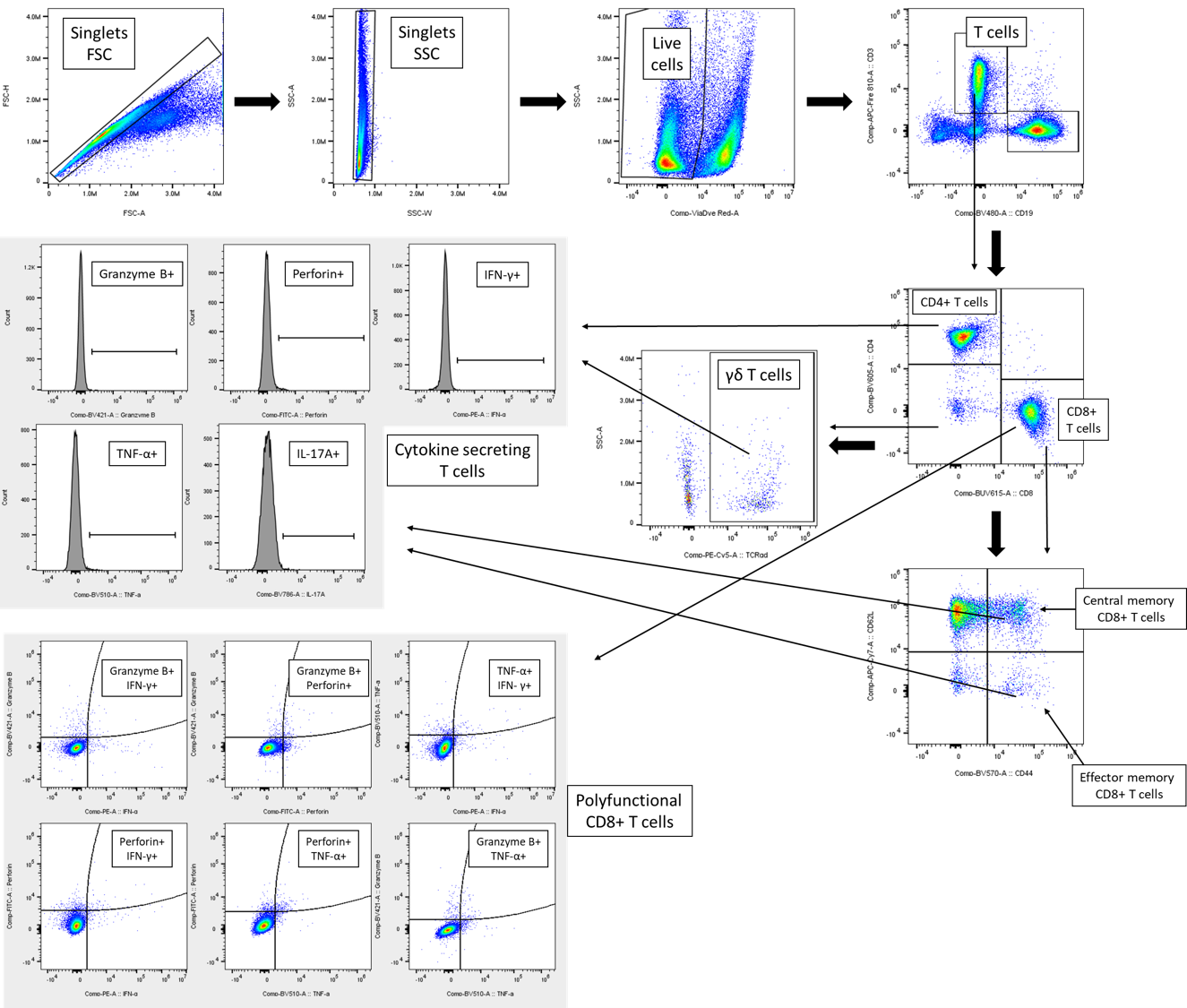

Supplement: S12 Fig — First live T cells were gated, followed by gating on CD4+, CD8+ or γδ T cells. CD8+ T cells were further separated by central memory or effector memory CD8+ T cells. For all cell populations intracellular cytokine production was measured. Additionally, CD8+ T cells were also analyzed for polyfunctionality, meaning the intracellular production of two or more cytokines. (TIF) [file ppat.1012764.s013.tif]
