# Supplementary material for: Immunopeptidomic MHC-I profiling and immunogenicity testing identifies Tcj2 as a new Chagas disease mRNA vaccine candidate
Source: PLoS Pathog. 2024 Dec 18;20(12):e1012764. doi: 10.1371/journal.ppat.1012764 (PMC11654963; doi:10.1371/journal.ppat.1012764)
Supplement: Attachment — Submitted filename: Reviewers comments PLOS Pathogens V2 FINAL CLEAN.docx [file ppat.1012764.s014.docx]

**Reviewers comments PLOS Pathogens Tcj2 manuscript**

**Part I - Summary**
Please use this section to discuss strengths/weaknesses of study, novelty/significance, general execution and scholarship.

Reviewer #1: While this manuscript provides some interesting data on (murine) Trypanosoma cruzi epitopes, there are major flaws which preclude publication in PLOS Pathogens. Please see section below.

We appreciate your feedback, and we will try to address and resolve your concerns and suggestions.

Reviewer #2: The authors present a very nicely linear story that starts from square 1 and ends at an an vivo proof of concept. It is very well written and very easy (because of their explanations and writing style) to understand why the authors made all of their decisions. It is always easy to anticipate what figure/data will come next at each stage of the manuscript. Overall, a very nicely written paper.

Thank you for your positive comments.

The authors are interested in characterising the MHC-1-presented peptide profile of T cruzi infected cells, with the aim of generating a CD8 T cell focused vaccine. They use robust techniques to identify candidate vaccine targets, eventually selecting the one and only protein candidate that was detected in multiple immunopeptidomic profile experiments. I can imagine that this experiment in itself may in some cases warrant a manuscript submission, however the authors take the next step and make an attempt to pre-clinically validate this vaccine strategy. They do so by generating an mRNA vaccine delivered by LNPs and assessing its immunogenicity in a mouse model. The significance of such an effort cannot be understated, given the several million people infected with T cruzi at present and lack of any effective clinical vaccine. The novelty of the strategy is underpinned by the identification of an entirely novel vaccine target for this parasite. Although the significance of this study is undermined by the lack of some important components of your typical chagas vaccine effort, those being:

A) some stronger evidence that the vaccine will be effective across different T cruzi stains/genotypes (the protein alignments are supportive, although unconvincing. e.g., expression profile differences could play a massive role),

Thank you for your feedback.

We agree with your suggestion that including the expression profiles of Tcj2 of different T cruzi stains/genotypes would strengthen the case for Tcj2 as a vaccine target. In fact, we do know that Tcj2 is well expressed in the Y strain (Li et al., 2016), as well as the Tulahuen based on our experiments. However, we agree that in the future, it will be important to further the evaluation of the expression profile of Tcj2 in different *T. cruzi* strains. In the meantime, we believe that our data suggests that Tcj2 is a promising vaccine target, and we addressed your concern in the discussion by adding the importance of future evaluations of Tcj2 in different *T. cruzi* strains.

B) the lack of any in vivo infection studies. Given the relatively weak immune response observed in 3/5 immunized mice (addressed again in part 2), I would be surprised to learn that this vaccine (at least at its current stage of development) provides anything more than the "partielle immunité" described by Brumpt over 100 years ago after the first chagas vaccine attempt (and also observed by basically everyone else who has tried). Still, these issues do not invalidate any of the findings in the manuscript itself. And one could envision that the follow up manuscript will have several in vivo infection studies, incorporate plenty of different adjuvants, and use several different parasite strains. Incorporating all of these into this manuscript would add years worth of time and effort, which is likely beyond the intended scope of this publication.

Thank you for your assessment, of which we agree. The primary aims of this first manuscript were to (i) demonstrate the utilization of immunopeptidomics to identify novel vaccine targets for Chagas disease, (ii) illustrate the potential of the mRNA platform to induce antigen-specific CD8+ T cell responses, and (iii) rapidly test new candidate antigens found through immunopeptidome screening. We agree that future work will include *in vivo* challenge models to test the efficacy of Tcj2 as a vaccine candidate, hopefully proving that this candidate vaccine antigen can induce significant protection against T. cruzi infection.

So indeed, this manuscript is appropriately novel, is executed well, and is scholarly enough; e.g., the authors appropriately reference several previous attempts to use this same strategy to identify vaccine candidates for other pathogens. I would also mention that the figure quality is for me generally excellent. The main weaknesses of the study relate to points A and B above, in addition to some more minor weaknesses (typographical, statistics, and a few missing controls).

Thank you for your valuable feedback. We hope that we have addressed your points A and B above.

Reviewer #3: This manuscript by Versteeg et al. describes vaccine antigen discovery work for Chagas disease, a protozoan parasite of significant global health impact for which no vaccine currently exists. The antigen discovery work leads to identification of a specific Chagas protein (Tcj2) as a potential key cytotoxic T cell-inducing antigen. This antigen discovery work is then validated by encoding of the Tcj2 protein into an mRNA-LNP vaccine and immunization of C57Bl/6 mice, resulting in humoral and antigen-specific CD8+ T cell responses to vaccination.

There is a general lack of work in the field of vaccine development for Chagas disease, with great needs for more studies identifying potential protective antigens against this parasite such as is included in this work. This manuscript is thus novel and of significant interest to the field and to global health efforts.

We appreciate your positive feedback!

The manuscript is generally well written, with various minor editorial issues. Overall this is a well thought out body of work with sound data interpretation and conclusions. The major defect of the work is the very small in vivo sample size combined with the apparent lack of any replicate in vivo study(ies). The entirety of the immune profiling of the lead identified Chagas antigen was carried out with a single study of n=5 all female C57Bl/6 mice at a single vaccine dose. The immune assays appear to be well designed and executed, and the resulting data certainly suggest support for the conclusions made, but this is insufficient to overcome the lack of in vivo sample size or any indication of technical study replicates.

Thank you for your feedback and suggestions. We acknowledge your comments that this study used a small in vivo sample size, with no replicates and using a single vaccine dose. However, we would like to highlight that this paper describes the results of a mouse study that was intentionally designed as a proof of principle study, to allow for maximum use of our resources to get immunogenicity data and draw some first conclusions with statistical significance. Therefore, by comparing the Tcj2 mRNA LNPs (n=5) with a negative LNP control group (n=5), we believe that we have sufficient data to demonstrate that our Tcj2 candidate vaccine antigen, identified by immunopeptidomics as a *T. cruzi* antigen that is presented on MHC-I molecules, can elicit a Th1-skewed immune response in mice when administered as a LNP formulated mRNA vaccine. Our aim in this study was not to determine optimal dose and immunization schedule for this mRNA vaccine to induce optimal immune responses or to assess induction of protective immunity. That will be assessed in future studies. In summary, we believe that our goal for this paper was to highlight the potential of Tcj2 mRNA LNPs as promising vaccine candidate for Chagas disease.

**Part II – Major Issues: Key Experiments Required for Acceptance**
Please use this section to detail the key new experiments or modifications of existing experiments that should be absolutely required to validate study conclusions.

Generally, there should be no more than 3 such required experiments or major modifications for a "Major Revision" recommendation. If more than 3 experiments are necessary to validate the study conclusions, then you are encouraged to recommend "Reject".

Reviewer #1: 1) There are no quality control measures implemented and shown for the immunopeptidomics experiment. The authors should e.g. provide figures on length distribution, binding properties, sequence clustering and retention time prediction for the identified peptides, all of which are common QC measurements in immunopeptidomics.

Thank you for your feedback. Besides the peptide properties shown in Table 1, we do also provide results on length distribution of the murine and *T. cruzi* peptides in Figure 2C and 2D. Additionally, to address your request, we added a table in the supplementary data which describes all the information we have on the *T. cruzi* peptides when the mass spec data was analyzed using PEAKS X. In this version of the PEAKS software, sequence clustering and retention time prediction are not available tools.

2) All Trypanosoma cruzi-derived peptides should be validated by comparison to synthetic peptide references.

Thank you for your recommendation to include a validation of identified peptides by comparing them to synthetic peptide references. We believe that in this case peptide validation may not be essential. We have assessed various papers from journals that have been published and where they did not perform such experiments (Blatnik et al., 2018; Draheim et al., 2017; Mou et al., 2015). We believe that the absence of synthetic peptide validation in our work does not invalidate our research findings. Moreover, in our manuscript where we focused for the majority on Tcj2, we identified six unique peptides in two independent immunopeptidomics experiments that all originated from this protein. This makes it very convincing that Tcj2 is presented as peptides on MHC-I during intracellular infection. Yet, we see the value of synthetic peptide references, and we will mention this in the limitation of our work section in the discussion. For future experiments we aim to run these synthetic peptide references to validate our peptides.

3) The conclusion that the vaccine elicits antigen-specific T cells is almost exclusively drawn from experiments evaluating SIINFEKL specific T cells, but not T cells directed against the previously identified epitopes. Here, e.g. loadable EasyMers for murine MHCs could be used to stain peptide-specific T cell populations.

Thank you for your feedback. We used the SIINFEKL sequence in the mRNA construct to evaluate the MHC-I processing and presentation using available tools, such as SIINFEKL-specific CD8+ T cells and SIINFEKL tetramers. Then, by stimulation of splenocytes from Tcj2 mRNA – immunized mice with recombinant Tcj2 protein, we observed the activation of Tcj2-specific CD8+ T cells (Figure 5D), as well as the production of cytotoxic cytokines and enzymes by CD8+ T cells (Figure 5E and Figure 6).

Finally, splenocytes from Tcj2 mRNA LNP immunized mice demonstrated killing of T*. cruzi* infected fibroblast in an *in vitro* killing assay. This suggests that Tcj2-specific CD8+ T cells recognized the *T. cruzi* infected cells through *T. cruzi* peptides presented on MHC-I. We believe that this is confirming that Tcj2 peptides are presented on MHC-I during host cell infection. We did not aim to confirm unique peptides in our assays, since these peptides sequences and lengths might change by conditions of the infected cells, MHC-I specificities and host species (McMurtrey et al., 2016). Loadable easymers are a very interesting tool to study peptide-specific CD8+ T cell populations, and we are considering to use them when we have fresh splenocytes available.

4) While Chronic Chagasic Cardiomyopathy is a human disease, all experiments in this manuscript were performed using murine model systems. The authors should strongly consider to provide evidence in the human setting by e.g. performing immunopeptidomics in Trypanosoma cruzi-infected human cell lines, using MHC-humanized mouse models (e.g. A2.DR1) and screening patient-derived PBMCs for responses against Trypanosoma cruzi epitopes identified in there.

Thank you for your suggestions. We agree that there is tremendous value in using human cell lines for T. cruzi research. However, we opted to focus on a murine model system because many of the biological questions about how T. cruzi infects host cells, modulates immune responses, and causes pathology, can be understood by using other mammalian cell lines including murine cells. Specifically, to start the immunopeptidomics program, a key criteria we used was that the cell line used would express robust levels of MHC-I on the cell surface, and that *T. cruzi* derived peptides would be presented by the cell line. Both these criteria were demonstrated previously by Kurup and Tarleton in the MC57G mouse fibroblast cell line(Kurup and Tarleton, 2014). Therefore, we opted to build on these findings with the MC57G cell line and identify which *T. cruzi* proteins are presented on MHC-I during infection. Future experiments will involve testing different tissues of T. cruzi in infected mice, as well as validation in human cell lines.

Reviewer #2: I have three "major issues"

The authors have a very limited description of the statistics used in each figure. In the methods section, they describe only the usage of a Mann Whitney U Test. It feels to me as though this manuscript is suffering from the multiple comparisons problem. Figures 5 and 6 are particularly problematic. The authors must either:

1. strongly justify why they are not correcting for multiple comparisons, or
2. perform a post-hoc analysis on the data, or
3. repeat the in vivo immunization study to increase the robustness of the dataset.

Simply put; it's very evident that almost all of the T cell data in these two figures is driven by 1 strongly responding mouse and partially by 1 moderately mouse. This needs to be addressed somehow. It doesn't invalidate the manuscript concept, but it needs to be addressed fairly via the above options.

Thank you for your feedback. We believe that the correct statistical analysis for the data in Figure 4 and Figure 5 is the Mann Whitney U test for the following reasons. A) in all the data, we are comparing only two groups, and asking the question whether there is a statistically significant difference between the Tcj2 mRNA LNP group and the empty LNP group. B) we did not assume normal distribution of the data. Therefore, we believe that a non-parametric T test, like the Mann Whitney U test, is the appropriate test.

We agree that there two mice in the Tcj2 mRNA LNP group that are stronger responders. However, many of the patterns that are observed in the data are not solely existent by one mouse. As an example, in Figure 5B all the mice in the Tcj2 mRNA LNP group were observed to have an increased percent (%) positive for SIINFEKL specific T cells compared to the control group, and this pattern is not present due to one mouse. Similarly, 4 out of 5 mice showed higher %-populations of CD8+ CD25+ compared to control group, as well as for CD8+ Perforin+. Since these patterns can be observed throughout our flow cytometry datasets, we think that we can attribute the observed patterns to the whole group.

Supplementary figure 5 is devoid of any positive controls for the presence/detection of the protein/target of interest in the various comparative cell lines. I understand what the authors mean to convey here, but given the already quite weak signal in the T cruzi lane, an approx. 3-fold downregulation of the protein in human cells relative to cruzi would prevent the band from showing up. We don't know if it is even expressed at all in the comparative lysates. For me this is too problematic - this figure should be removed unless the proper positive controls can be acquired (e.g., anti-human DnaJ).

We appreciate your suggestions, and we understand that a control demonstrating the presence of e.g. human DnaJ in the human HEK293T lysate would be useful. We went ahead and purchased an antibody that can detect DNAJA4, which is a human DnaJ protein that has the highest sequence identity to *T. cruzi*’s Tcj2 of all human DnaJs. We used this anti-human DNAJA4 antibody (ThermoFisher, Cat# PA5-65311) to confirm expression of human DnaJ in the human HEK293T lysate samples (Supplementary Figure 6). The reactivity to human DnaJ but not to T. cruzi or mouse DnaJ demonstrates the reduced chance or cross-reactivity between different species. We mentioned these results in line 308 in the results.

Figure 2B is very weak. If this plot was represented instead as the original FACS histograms, I assume it would look like there was no change from control to experimental. The call out of Fig 2B in the text also feels somewhat out of order. And finally, the references (51 and 52) mentioned in the discussion seem to have substantially more robust datasets that suggest that MHC is downregulated by Tc infection. I think this discrepancy could easily come down to a technical / cell line issue but I think its impossible to use this (evidently not statistically significant) piece of data to make any conclusions. I would recommend either:

1. repeating this assay using a more robust technique (e.g., qPCR or a commercially reliable antibody instead of this in-house antibody), or
2. omitting this piece of data

Thank you for your feedback. The M1/42 mAb is a well-characterized antibody that binds to mouse MHC-I (Stallcup et al., 1981). We repeated the experiment to show the increase of MHC-I expression by murine MC57G fibroblasts during T. cruzi infection using a commercially available M1/42 mAb conjugated to Alexa Fluor 488. We executed the experiment using the previous methodology, but in this case, we used the Attune NxT flow cytometer. The results can be observed in Figure 2B and Supplementary Figure 8. We are pleased to report that the previous results reported were confirmed, as the results showed that MHC-I expression is significantly increased in T. cruzi infected MC57G cells compared to uninfected cells. Additionally, we did more literature review and came across a publication in which they reported multiple murine cell types that upregulate MHC-I expression upon T. cruzi infection (Stryker and Nickell, 1995). We added this reference to our discussion for completion.

Reviewer #3: 1. The in vivo immunogenicity study is underpowered and not replicated. This is a major drawback of the study. At a minimum a replicate study should be completed, which would at least bring the sample number to n=10 for the vaccinated mice. More appropriately, to meet current scientific standards for rigor and reproducibility, an additional study would include groups of 8-10 vaccinated mice, half male and half female, with a better control (LNP-formulated off-target mRNA) for comparison, and possibly comparing/contrasting the responses to multiple different vaccine doses rather than just one very high dose.

Thank you for your feedback. To clarify, mice received two immunizations of the Tcj2 mRNA LNPs, and we changed the wording throughout the manuscript to make this clearer. Additionally, our goals were initially to use Ovalbumin mRNA as an off-target mRNA construct as control, but our IACUC animal committee recommended to use empty LNPs due to the possible strong reaction to the immunogenic Ovalbumin.

While we understand that the number of mice in each study group is on the smaller side, we believe that as a pilot study, this dataset is sufficient to highlight that the Tcj2 mRNA LNPs is a promising vaccine candidate against Chagas disease. Furthermore, we designed the mouse study to allow for maximum use of our resources to get immunogenicity data and draw some first conclusions with statistical significance. Comparing the Tcj2 mRNA LNPs (n=5) with a negative LNP control group (n=5), we believe that we have sufficient data to demonstrate that our Tcj2 mRNA can successfully be translated in vivo, eliciting a Th1-skewed immune response in mice.

We understand the reviewers' request for a larger set of *in vivo* data. However, following the ethical guidelines for animal research, we aimed not repeating the immunogenicity study to reduce the # of animal needed. Instead, we hope to use this first set of data and results to pursue funding opportunities and get funding for additional studies as well as a challenge animal study in which one arm of the study will serve the dual purpose of repeating the immunogenicity study. In future studies we also aim to perform a more in depth immune analysis, and we will include male and female mice as well.

We believe that for this manuscript, our goal was to highlight the potential of Tcj2 mRNA LNPs as promising vaccine candidate for Chagas disease. This goal was reached with the size of the animal study and the performed experiments.

2. The immunopeptidomics studies are conducted using T. cruzi infected mouse fibroblast cells. Given that the ultimate goal of this vaccine development effort is to develop vaccines for human populations, why were human cells not used? Ideally human cells representing a variety of HLA class 1 haplotypes? The choice of this cell line is neither discussed nor justified, but would appear to be of substantial import for the validity of the results. This choice must be justified, and/or further data collected to validate the results (i.e., should Tcj2 peptides show up prominently as class I HLA presented peptides on human cells, in addition to the mouse data shown, that would be substantial enhancement of the key result that this may be an excellent antigen for induction of T. cruzi-reactive CTLs across species).

Thank you for your suggestions. We agree that there is tremendous value in using human cell lines for *T. cruzi* research. This was also highlighted by reviewer 1 .(see above). Briefly, we opted to focus on a murine model system because many of the biological questions about how T. cruzi infects host cells, modulates immune responses, and causes pathology, can be understood by using other mammalian cell lines including murine cells. Specifically, to start the immunopeptidomics program, a key criteria we used was that the cell line used would express robust levels of MHC-I on the cell surface, and that *T. cruzi* derived peptides would be presented by the cell line. Both these criteria were demonstrated previously by Kurup and Tarleton in the MC57G mouse fibroblast cell line(Kurup and Tarleton, 2014). Therefore, we opted to build on these findings with the MC57G cell line and identify which *T. cruzi* proteins are presented on MHC-I during infection. Future experiments will involve testing different tissues of T. cruzi in infected mice, as well as validation in human cell lines.

3. Very scant details are provided for the mRNA vaccine candidate that was engineered to express Tcj2. For example, what UTRs were used? These affect modified mRNA vaccines significantly. A statement is made that the RNA sequence was optimized for translation – how was this done, using what algorithm? What LNP system was used for encapsulation? Were there any measures of RNA integrity? It’s appreciated that basic particle size and PdI is included as part of vaccine characterization as size and charge are key parameters for cellular uptake and thus vaccine efficacy. But more information – ideally the entirety of the RNA sequence – must be included for appropriate interpretation of the data.

Thank you for your feedback. The mRNA UTRs are from human β-globin. The complete sequence was codon optimized by the RNAcore at the Houston Methodist Research Institute using their proprietary algorithm which considers dozens of key factors of gene transcription and translation. The codon optimizated mRNA sequence, as well as the plasmid sequence used for in vitro transcription of the mRNA, and property of the RNAcore.

The mRNA was formulated in lipid nanoparticles (LNPs) using a commercial kit (Genvoy, Precision Nanosystems) using a microfluidic benchtop system (NanoAssemblr, Precision Nanosystems), all described in the methods section under “mRNA vaccine formulation in Lipid Nanoparticles (LNPs)”. Validation of mRNA integrity was conducted through agarose gel electrophoresis, while protein translation across various cell lines was assessed via western blot analysis. We have added an electrophoresis gel showing the integrity of the Tcj2 mRNA construct in supplementary Figure 9.

**Part III – Minor Issues: Editorial and Data Presentation Modifications**
Please use this section for editorial suggestions as well as relatively minor modifications of existing data that would enhance clarity.

Reviewer #1: (No Response)

Reviewer #2: There are a moderate number of typographical errors throughout. Some of the standouts are SIINFELK vs SIINFEKL (line 216) and the location of the SIINFEKL motif being at the 3' (the correct one according to Fig 3) vs the 5' end of the mRNA construct (line 292). The manuscript should be proofread.

Thanks for the suggestions. We have changed the typographical errors throughout the manuscript.

The manuscript opens several lines of questions. I am unclear as to why/how the apparent majority of the T cruzi MHC-presented proteins are intracellular. The hypothesis that strikes me is based on cell death, especially since their top candidate is an HSP. Is it possible that only a small fraction of the infected cells are actually presenting these epitopes? The fraction of cells that happen to have some dead T cruzi cells within them? That may also explain the marginal (10% above control) phenotype observed in the final figure. I would recommend the authors to address this point in greater detail in the discussion.

Thank you for your feedback. We do not believe that heat shock proteins are specifically related to cell death, and that their identification by immunopeptidomics indicates the death of T. cruzi parasites. Heat shock proteins like DnaJs are involved in (re)folding of proteins and are expressed in both trypomastigote and amastigote stages of the parasite (Paba et al., 2004), and there are indications that DnaJs are secreted by T. cruzi as well (Watanabe Costa et al., 2020). Also, DnaJ proteins were prevalent in the secretome of trypomastigotes transforming to amastigotes by incubation in low pH (Mandacaru et al., 2019). Based on findings from other research, we believe that DnaJ proteins are secreted and can become available to MHC processing and presentation while the T. cruzi parasites are alive in the host cell.

I assume by "titer" the authors mean "end point titer" - please clarify

Thank you for the feedback. Yes we meant “end point” antibody titer with titer. We improved the manuscript by always mentioning “antibody titer” instead of just titer, and we mentioned that the antibody titers represents end point titers in the methods section.

In some figures, e.g., Fig 2B, there is no description in the figure legend highlighting what the error bars mean. How many independent experiments? etc.

Thanks for the suggestion, we will add the information on the error bars to all figures. Figure 2B consists of six technical replicates. The experiment that yielded the results for this figure has been repeated per request from one of the reviewers, the figure has been updated and results incorporated in the manuscript.

Reviewer #3: 1. Lines 174-209 discuss the similarity/conserved nature of Tcj2 proteins from related trypanosomes, concluding that the use of this antigen in vaccination may induce cross-protection against other trypanosomic infections. Much of this section is speculative and more appropriate for the Discussion section than the Results section. This reviewer recommends condensing this section in the Results and moving some content to the Discussion section.

Thank you for your suggestion. While we agree that parts of the text are explaining the results and could be seen as discussion, much of this section is referring to results from protein sequence analysis and explaining the specific comparisons made between sequences of different T. cruzi strains and different species. To avoid repetitive text and avoid making the discussion extra lengthy, we improved parts of the discussion to address this comment as well as the comment pointed out in point 3.

2. Lines 194-195 state that Tcj2 and human DnaJ have 43% identity and 60% similarity, and the statement is made that this represents sufficient difference to make overlapping T cell epitopes unlikely resulting in a low risk of autoimmunity. Later statements are made about ~70% identity conservation between Tcj2 sequences in different trypanosomes being potentially sufficient for cross-protection. Clearly there is a difference between 43% and 70% similarity, but is there a known cutoff/what evidence is available to say that one is likely non-cross-reactive and the other potentially cross-reactive?

Thank you for your question. While the %-identity gives a general indication how identical the amino acid sequences are between proteins, it is most relevant if there are (or not) long enough identical 8-15-mer amino acid sequences that will allow presentation of identical peptides between the species that are compared. Since there are hardly stretches of 8 (or more) amino acids that are identical between the species, cross-reactivity due to complete sequence identity is not expected.

Besides the explanation of this in line 195-202 and the Supplementary Figure 2 that illustrates the identity between Tcj2 and ortholog sequences, we added extra text to the discussion for clarity to the reader.

3. Discussion – the Discussion is lengthy and reads as a blow-by-blow of the Results, with a bit of additional discussion that lacks some key content. A brief discussion of the strengths and weaknesses of this study, particularly in context of the field (what does this study add to existing research) is lacking and should be added. What would the proposed final vaccine product be? Would it be focused on Tcj2 alone as an antigen, or more likely in combination with other potentially key T cruzi antigens? If so, which might be good combinations, with literature to support, and would follow-on studies be necessary to determine optimal combinations? What is known about correlates of protection against T cruzi, beyond what is briefly mentioned about IgG2? Are in vivo challenge models available that would be sufficient for such studies?

Thank you for your suggestions. We rewrote parts of the discussion to improve the structure. We added a section in the discussion that mentions the limitations (weaknesses) of the study. Furthermore, we agree that we did not discuss the context of Tcj2 in the field of Chagas disease vaccines. In this manuscript we hope to convey that immunopeptidomics is a powerful tool to analyze peptides presented on host cells from intracellular parasites such as T. cruzi, and that Tcj2 is a promising vaccine target that induces a cytotoxic T cell response in mice when formulated as a Tcj2 mRNA LNP vaccine. We aim to perform additional studies on Tcj2 as a vaccine target and test its efficacy in future studies including a mouse challenge model. Here, we will expand and add additional description of Tcj2 and its role in the field of Chagas disease vaccine development.

4. Statistics: why is a non-parametric test used uniformly for all statistical analyses? While nonparametric tests avoid assumptions about data normality, their use reduces statistical power relative to appropriate parametric tests. Various immune readouts – such as log-normalized serum Ab data – reliably are normally distributed and thus the use of nonparametric tests (particularly when used on an already underpowered n=5 sample size) is not necessary.

We appreciate your suggestion. The serum IgG titer data has not been log-normalized, but the y-axis is shown in log-format. Therefore, we cannot assume that the data is normally distributed. We therefore believe we should stay with the non-parametric Mann-Whitney U test for our statistical analysis.

5. Tcj2 is a candidate vaccine antigen. Not a candidate vaccine. Only the Tcj2-expressing mRNA-LNP vaccine is a vaccine. Please edit the language throughout the manuscript appropriately.

Thank you for your feedback. We corrected this language throughout the manuscript.

6. Line 48 contains an unnecessary “a”.

Thank you, we removed the unnecessary “a”.

7. Line 57: unclear whether the 20-30% with CCC are a subset of the 30-40% of cases that develop chronic disease, or a subset of total Chagas cases. Please clarify.

Thank you for your question. The 20-30% with CCC is not a subset but a total, we clarified this in the manuscript.

8. Line 67: the proposed vaccine would presumably prevent, rather than cure, Chagas disease, correct? If there is a therapeutic potential use for the proposed vaccine, rather than a standard prophylactic use, please make this clear as it would greatly affect the use cases for the vaccine.

Thank you for your question. At this stage we do not specify whether the Tcj2 mRNA LNP vaccine candidate will be prophylactic or therapeutic. Additional studies including challenge studies need to be performed, both with prophylactic and therapeutic use of the vaccine candidate, to investigate the effectiveness of the vaccine at different stages of infection. Furthermore, while a preventative vaccine could help travelers and people living in endemic regions, a therapeutic vaccine could help people currently with Chagas disease delaying or stopping the progression of disease.

9. Line 82: need to add “proteins” after MHC.

Thank you for the suggestion, we added the word proteins for clarity.

10. Line 85: “superior sensitivity” relative to what? Please clarify.

Thank you for your question. The identification of MHC presented peptides at a superior sensitivity is relative to the previous versions of mass spectrometers, for which it is very challenging to identify relatively small peptides due to technological limitations.

11. Lines 90-92: sentence is convoluted

Thank you, we improved this sentence.

12. If a major issue with the design of vaccines against Chagas is a lack of known protective epitopes, please clearly state this along with any prior knowledge in the area. Any prior knowledge is not clearly expressed in the introduction and should be.

Thank you for your comment. For the design of Chagas disease vaccines, a lack of known protective epitopes is not necessarily the issue, but more importantly it is challenging to find T. cruzi antigens that have protective properties. Protective CD8+ T cell epitopes can broadly differ between allotypes of MHC-I of mouse and human, and are therefore less of a focus than complete antigens. Therefore, we also created a mRNA vaccine construct that encodes for the complete Tcj2 protein.

13. Lines 97-98: Most human studies of immune responses to Covid mRNA vaccines to this reviewer’s knowledge do not indicate strong CTL responses. Please add citations for this statement.

Thank you for your suggestion, we added citations that support this statement.

14. Lines 98-100: The cited work here is a pre-Covid review article by many of the same authors – given the huge leap forward in understanding of immunogenicity of modified mRNA-LNP vaccines since this time, a more recent reference would be more appropriate.

Thank you for this feedback, we added more recent references on this topic.

15. Line 109 – Tcj2 is a candidate vaccine antigen, not a candidate vaccine.

Thank you for the suggestion, we corrected this in the manuscript.

16. Line 115 – Tcj2 expressing mRNAs formulated in LNPs.

Thank you for the suggestion, we corrected this in the manuscript.

17. Line 135 – two replicate(?) immunopeptidomics experiments. If there were any technical differences between these please clarify, and if not, state that they are technical replicates.

Thank you for your question. Yes, the two experiments are technical replicates. We clarified this in the manuscript.

18. Figure 2: How many peptides that were immunoaffinity purified and made it through the LC/MS/MS process were not identifiable? If none, state so.

Thank you for your question. All the peptides that were run through the mass spectrometer were identified. Besides the murine self-peptides and the T. cruzi peptides, there were a couple of peptides that were identified from bovine proteins, which is originating from the fetal bovine serum that is supplemented in the culture media.

19. Line 190: what are TcI, TcII, and TcVI from TriTryDB? Please define or reword. If nonimportant for the conclusions, move to methods.

Thank you for your comment. We reworded the sentence for improved readability to: “When comparing the Tcj2 sequence between the different genetic groups of *T. cruzi*, it was observed that Tcj2 is very conserved (Supplementary Figure 1).”

20. Line 195: comparisons are being made between human DnaJ and T. cruzi DnaJ-typeTcj2 genes/proteins, no? The statement as is implies that the comparisons are between the full genomes of the two rather than just these particular genes. Please reword for clarity.

Thank you for your question. The comparison is indeed between the protein sequences only. We improved the text in line 195-197 to clarify this.

21. Lines 199-200: please briefly summarize the key conclusion in reference 42 that backs this statement up. Is there a similarity threshold below which self-interactions can be safely assumed to be negligible?

Thank you for your feedback. Upon review of the current reference #42, we agreed that this reference was not as relevant. We added a more appropriate reference instead (Tagliamonte et al., 2023). Furthermore, we reviewed the mechanisms of molecular mimicry in detail, such that we would be clearer in the manuscript. As you mention, in some cases, structural similarity of peptide sequences can potential cause self-interactions, even though amino acids sequences of peptides are not identical. There is no threshold for similarity that can be applied to each peptide to predict self-interactions. For a protein the %-similarity is calculated by taking the structural properties of the amino acids into account. For peptides, these structural properties need to be considered in combination of the anchor positions of the peptide, which where each peptide binds to the MHC-I groove. The latter has not been done by us and we consider to be outside the scope of the project. Since this is a limitation of our work, we added text in the discussion to mention this.

22. Line 214: Tcj2-expressing mRNA vaccine.

Thank you for the suggestion, we corrected this in the manuscript.

23. While the use of a FLAG tag and SIINFEKL sequence are understandable for validation purposes, these are typically not considered to be appropriate for clinical vaccines as such tags have been known to affect expressed protein structure and stability. A brief mention, even in the Discussion, of an intent to remove these for IND-enabling studies would likely be appropriate.

Thank you for the suggestion. We have incorporated a sentence stating that these tags will be removed if the vaccine construct reaches a critical path towards their use for clinical evaluations in the discussion.

24. Line 258 – what has been demonstrated is not yet the ability of Tcj2-encoding mRNAs to induce the establishment of new antigen-specific CD8+ T cell populations, but the ability of mRNA-expressed protein to trigger existing antigen-specific CD8 T cells to respond. Please reword to imply the latter rather than the former.

Thank you for the suggestion, we corrected this in the manuscript.

25. Line 258: Tcj2-expressing (or encoding) mRNAs were formulated in lipid nanoparticles (LNPs) for further in vivo assessment of Tcj2 as a vaccine antigen (or Tcj2-expressing mRNA-LNPs as a vaccine candidate). The vaccine candidate is the mRNA-LNP combination, not the RNAs alone.

Thank you for the suggestions, we corrected them in the manuscript.

26. Line 260: why were empty LNPs used as a control, rather than nonsense RNA of similar size to the Tcj2 mRNAs, or better yet, an off-target mRNA such as OVA-expressing mRNA? As noted in the data, LNPs do not form properly/uniformly if empty of RNA and may not be the most ideal control. If a follow-up or replicate in vivo study is performed, this could be easily added/changed.

Thank you for your suggestions. Generally, when we only perform immunogenicity and look at antigen-specific responses, we do not add an antigen in the control group (for example, for recombinant protein vaccines with adjuvant, the control group contains only adjuvant). We applied the same process in this case, because we do not anticipate an antigen-specific response.

27. Lines 263-265: Temperature of vaccine freezing should be mentioned (-80C?). Freezing was followed by thawing before particle size measurements by DLS, correct? If so, the term freeze/ thaw should be correctly applied.

Thank you for your suggestion. We improved the wording to clarify that LNPs were freeze/thawed before analysis.

28. Empty LNPs polydispersity index decreased significantly upon freeze/thaw? Do explain.

Thank you for the question. Empty LNPs are not as stable and uniform formed as loaded LNPs due to the absence of mRNA. Consequently, when they are subject to stress, such as a freeze/thaw cycle, unstable LNPs do not survive and disappear. This results in the enrichment of more stable and better formed LNPs in a sample. This is visible through the improvement of the polydispersity of the empty LNP sample after they went through a freeze/thaw cycle.

29. Line 272: “The PkA of LNPs has been previously shown to be a determining factor….”

Thank you, we improved the grammar of this sentence.

30. Figure 4 panel A – column titled “LNPs” should more accurately be “LNP cargo”

Thank you, we implemented this improvement in the figure.

31. Figure 4 panel B – there appear to be a large species (<1000 nm) present for the empty LNPs. Can you speculate what this population is and what if any affects it may have upon injection into mice?

Thank you for your feedback. LNPs that contain no mRNA have a less uniform size due to the absence of negatively charged mRNA that helps the uniform and stable formation of the LNPs. This can result in LNPs with a less uniform shape, as observed in the polydispersity index in Figure 4A. Furthermore, in Dynamic Light Scattering, particles that are larger are observed with a higher intensity compared to smaller particles. This effect skews the intensity of larger particles, while these populations are rather small.

LNPs that have a size that is larger than the typical 80-100 nm diameter, might be prone to increased phagocytosis by antigen presenting cells like DCs and macrophages. This could lead to an activation of the innate immune system at the site of the immunization. However, since we believe the population of large LNPs in the vaccine in very small, this is of minimal concern for this immunogenicity study. Moreover, since we are only looking at Tcj2-specific immune responses, we do not believe that our data would be affected by a possible activation of non-specific innate immune responses.

32. Figure 5: The figure contains panels with linear and with log axes. Please define what the bars indicate for each – on log axes do they appropriately indicate geometric means and geometric stdevs? For linear axes plots, do they indicate arithmetic means and stdevs?

Thank you for your comment. We agree that we did not clarify well what the bars and error bars were indicating. We have added this information in the legends of each figure.

33. Figure 5 panel C – the rightmost bar (IgG2c serum Tcj2-binding IgG titers) shows error bars much larger than/more than spanning the displayed 4 datapoints. First – why are there just 4 datapoints rather than 5, given that there were 5 immunized mice and 5 datapoints on the other bars? Second – why are the error bars so big? Are these arithmetic stdevs rather than geometric stdevs? Please investigate for all bars in this panel.

Thank you for your feedback. We realized that one datapoint fell outside the range of the y-axis, and we corrected the plot by increasing the upper limit of the y-axis. Furthermore, the geometric means and 95% confidence intervals of the graph are shown in the graph, and we added this information in the figure legend for clarity.

34. Figure 5 panel E: IFNg expressing CD8+ T cell populations are surprisingly low, particularly given the typically good CD8/IFNg responses in B/6 mice. Can you explain?

Thank you for your feedback. While we agree that of the two mouse stains BALB/c and C57BL/6, the latter is more skewed towards Th1, we do not think that our IFN-g responses are out of the ordinary. First, our observations are in line with previous published work in which we immunized C57BL/6 mice with Tc24 mRNA LNPs followed by in vitro restimulation of splenocytes and flow cytometry analysis (Poveda et al., 2023). Second, we perform the restimulation of the splenocytes in vitro using recombinant protein and not with peptides. For proteins to activate CD8+ T cells, they need to be processed by antigen-presenting cells first and presented on MHC-I. While this can be very efficient for peptide restims, this can be less efficient for proteins since their natural tendency for MHC-II presentation. While CD8+ T cell activation can still be measured successfully through protein restims, the magnitude of the CD8+ T cell response might be lower compared to peptide restims. Still, even when peptide pools are used the specific IFN-g CD8+ T cell response may show a variety in magnitude based on the study (examples here: (Liu et al., 2022; Peng et al., 2022)).

35. Figure 5 flow data – please indicate on y-axes what the indicated population %s are based on. i.e., % of live CD3+CD4+ T cells, or similar.

Thank you for the suggestion. We improved the y-axis of the flow data of figure 5 based on your recommendation.

36. Line 323 please delete or clearly define the descriptor “robust”.

Thank you for your suggestion, we removed the term “robust”.

37. Line 326: why was baseline titer set to 67 and why is this an appropriate LOD?

Thank you for your question. The antibody titer is an arbitrary value that represents the dilution factor of the sera where the OD value of the samples is equal or lower than the titer cutoff value. In our ELISAs, we start all our sera dilutions at a start dilution of 1:300 followed by 3-fold dilutions. When the OD value of a sample does need reach the titer cutoff value at the first dilution of 1:300, we report the value of the theoretical dilution below that, which is 1:67. This is considered the baseline titer. We have used this analysis in several projects(Pollet et al., 2021; Poveda et al., 2023; Villanueva-Lizama et al., 2020).

38. Lines 334-335: sentence is confusing.

We changed the sentence for improved readability.

39. Line 344: “…by CD8+ T cells when exposed to antigen…”

We changed the sentence for improved readability.

**References**

Blatnik, R., Mohan, N., Bonsack, M., Falkenby, L.G., Hoppe, S., Josef, K., Steinbach, A., Becker, S., Nadler, W.M., Rucevic, M., Larsen, M.R., Salek, M., Riemer, A.B., 2018. A Targeted LC-MS Strategy for Low-Abundant HLA Class-I-Presented Peptide Detection Identifies Novel Human Papillomavirus T-Cell Epitopes. PROTEOMICS 18, 1700390. https://doi.org/10.1002/pmic.201700390

Draheim, M., Wlodarczyk, M.F., Crozat, K., Saliou, J.-M., Alayi, T.D., Tomavo, S., Hassan, A., Salvioni, A., Demarta-Gatsi, C., Sidney, J., Sette, A., Dalod, M., Berry, A., Silvie, O., Blanchard, N., 2017. Profiling MHC II immunopeptidome of blood-stage malaria reveals that cDC1 control the functionality of parasite-specific CD4 T cells. EMBO Mol. Med. 9, 1605–1621. https://doi.org/10.15252/emmm.201708123

Kurup, S.P., Tarleton, R.L., 2014. The Trypanosoma cruzi flagellum is discarded via asymmetric cell division following invasion and provides early targets for protective CD8^+^ T cells. Cell Host Microbe 16, 439–449. https://doi.org/10.1016/j.chom.2014.09.003

Li, Y., Shah-Simpson, S., Okrah, K., Belew, A.T., Choi, J., Caradonna, K.L., Padmanabhan, P., Ndegwa, D.M., Temanni, M.R., Bravo, H.C., El-Sayed, N.M., Burleigh, B.A., 2016. Transcriptome Remodeling in Trypanosoma cruzi and Human Cells during Intracellular Infection. PLOS Pathog. 12, e1005511. https://doi.org/10.1371/journal.ppat.1005511

Liu, J., Budylowski, P., Samson, R., Griffin, B.D., Babuadze, G., Rathod, B., Colwill, K., Abioye, J.A., Schwartz, J.A., Law, R., Yip, L., Ahn, S.K., Chau, S., Naghibosadat, M., Arita, Y., Hu, Q., Yue, F.Y., Banerjee, A., Hardy, W.R., Mossman, K., Mubareka, S., Kozak, R.A., Pollanen, M.S., Martin Orozco, N., Gingras, A.-C., Marcusson, E.G., Ostrowski, M.A., 2022. Preclinical evaluation of a SARS-CoV-2 mRNA vaccine PTX-COVID19-B. Sci. Adv. 8, eabj9815. https://doi.org/10.1126/sciadv.abj9815

Mandacaru, S.C., Queiroz, R.M.L., Alborghetti, M.R., de Oliveira, L.S., de Lima, C.M.R., Bastos, I.M.D., Santana, J.M., Roepstorff, P., Ricart, C.A.O., Charneau, S., 2019. Exoproteome profiling of Trypanosoma cruzi during amastigogenesis early stages. PLoS ONE 14, e0225386. https://doi.org/10.1371/journal.pone.0225386

McMurtrey, C., Trolle, T., Sansom, T., Remesh, S.G., Kaever, T., Bardet, W., Jackson, K., McLeod, R., Sette, A., Nielsen, M., Zajonc, D.M., Blader, I.J., Peters, B., Hildebrand, W., 2016. Toxoplasma gondii peptide ligands open the gate of the HLA class I binding groove. eLife 5, e12556. https://doi.org/10.7554/eLife.12556

Mou, Z., Li, J., Boussoffara, T., Kishi, H., Hamana, H., Ezzati, P., Hu, C., Yi, W., Liu, D., Khadem, F., Okwor, I., Jia, P., Shitaoka, K., Wang, S., Ndao, M., Petersen, C., Chen, J., Rafati, S., Louzir, H., Muraguchi, A., Wilkins, J.A., Uzonna, J.E., 2015. Identification of broadly conserved cross-species protective *Leishmania* antigen and its responding CD4 ^+^ T cells. Sci. Transl. Med. 7. https://doi.org/10.1126/scitranslmed.aac5477

Paba, J., Ricart, C.A.O., Fontes, W., Santana, J.M., Teixeira, A.R.L., Marchese, J., Williamson, B., Hunt, T., Karger, B.L., Sousa, M.V., 2004. Proteomic Analysis of Trypanosoma cruzi Developmental Stages Using Isotope-Coded Affinity Tag Reagents. J. Proteome Res. 3, 517–524. https://doi.org/10.1021/pr034075o

Peng, L., Renauer, P.A., Ökten, A., Fang, Z., Park, J.J., Zhou, X., Lin, Q., Dong, M.B., Filler, R., Xiong, Q., Clark, P., Lin, C., Wilen, C.B., Chen, S., 2022. Variant-specific vaccination induces systems immune responses and potent in vivo protection against SARS-CoV-2. Cell Rep. Med. 3, 100634. https://doi.org/10.1016/j.xcrm.2022.100634

Pollet, J., Chen, W.-H., Versteeg, L., Keegan, B., Zhan, B., Wei, J., Liu, Z., Lee, J., Kundu, R., Adhikari, R., Poveda, C., Villar, M.J., Leao, A.C. de A., Rivera, J.A., Momin, Z., Gillespie, P.M., Kimata, J.T., Strych, U., Hotez, P.J., Bottazzi, M.E., 2021. SARS‑CoV-2 RBD219-N1C1: A yeast-expressed SARS-CoV-2 recombinant receptor-binding domain candidate vaccine stimulates virus neutralizing antibodies and T-cell immunity in mice. Hum. Vaccines Immunother. 0, 1–11. https://doi.org/10.1080/21645515.2021.1901545

Poveda, C., Leão, A.C., Mancino, C., Taraballi, F., Chen, Y.-L., Adhikari, R., Villar, M.J., Kundu, R., Nguyen, D.M., Versteeg, L., Strych, U., Hotez, P.J., Bottazzi, M.E., Pollet, J., Jones, K.M., 2023. Heterologous mRNA-protein vaccination with Tc24 induces a robust cellular immune response against Trypanosoma cruzi, characterized by an increased level of polyfunctional CD8+ T-cells. Curr. Res. Immunol. 4, 100066. https://doi.org/10.1016/j.crimmu.2023.100066

Stallcup, K.C., Springer, T.A., Mescher, M.F., 1981. Characterization of an anti-H-2 monoclonal antibody and its use in large-scale antigen purification. J. Immunol. Baltim. Md 1950 127, 923–930.

Stryker, G.A., Nickell, S.P., 1995. *Trypanosoma cruzi:* Exposure of Murine Cells to Live Parasites *in Vitro* Leads to Enhanced Surface Class I MHC Expression Which Is Type I Interferon-Dependent. Exp. Parasitol. 81, 564–573. https://doi.org/10.1006/expr.1995.1150

Tagliamonte, M., Cavalluzzo, B., Mauriello, A., Ragone, C., Buonaguro, F.M., Tornesello, M.L., Buonaguro, L., 2023. Molecular mimicry and cancer vaccine development. Mol. Cancer 22, 75. https://doi.org/10.1186/s12943-023-01776-0

Villanueva-Lizama, L.E., Cruz-Chan, J.V., Versteeg, L., Teh-Poot, C.F., Hoffman, K., Kendricks, A., Keegan, B., Pollet, J., Gusovsky, F., Hotez, P.J., Bottazzi, M.E., Jones, K.M., 2020. TLR4 agonist protects against Trypanosoma cruzi acute lethal infection by decreasing cardiac parasite burdens. Parasite Immunol. 42, e12769. https://doi.org/10.1111/pim.12769

Watanabe Costa, R., Batista, M.F., Meneghelli, I., Vidal, R.O., Nájera, C.A., Mendes, A.C., Andrade-Lima, I.A., da Silveira, J.F., Lopes, L.R., Ferreira, L.R.P., Antoneli, F., Bahia, D., 2020. Comparative Analysis of the Secretome and Interactome of Trypanosoma cruzi and Trypanosoma rangeli Reveals Species Specific Immune Response Modulating Proteins. Front. Immunol. 11.
