# Supplementary material for: Immunopeptidomic MHC-I profiling and immunogenicity testing identifies Tcj2 as a new Chagas disease mRNA vaccine candidate
Source: PLoS Pathog. 2024 Dec 18;20(12):e1012764. doi: 10.1371/journal.ppat.1012764 (PMC11654963; doi:10.1371/journal.ppat.1012764)
Supplement: Attachment — Submitted filename: Reviewers comments PLOS Pathogens FINAL.docx [file ppat.1012764.s015.docx]

**Reviewers comments PLOS Pathogens Tcj2 manuscript**

This paper is on a very important topic (the first identification of a specific protein as a vaccine target for T cruzi (eliciting an anti-cruzi CD8+ T cell response). However, taking stock of all reviewer comments (including a reviewer who refused to review without the requested changes), it appears that the authors were not responsive to reviewer requests (which are quite reasonable and which will clearly impart robustness to the paper). Some of these comments that were not addressed at all are below:

Thank you for your feedback. While we incorporated many of the reviewers suggestions in the previous revisions of our manuscript, we acknowledge that specific requests regarding the peptide validation and a repeat of the mouse *in vivo* study were not addressed. We have now completed these additional experiments and included the results in the revised manuscript to further enhance its robustness. Specifically, the peptides derived from Tcj2 were validated by comparing their mass spectrometry spectra with synthetic Tcj2 peptides. Additionally, the mouse *in vivo* study was repeated to confirm reproducibility. We believe these revisions align with the reviewers' requests and have strengthened the manuscript.

1) immunopeptidomics was done to identify the specific epitope (but no validation with synthetic peptides was even attempted despite reviewer concerns - this is not a hard expt to do, it is a key experiment to do and it cannot be argued away)

Thank you for the suggestion. We have analyzed synthetic peptides from Tcj2 and compared their mass spectrometry spectra with the Tcj2-peptides found in the immunopeptidomics experiments, confirming that these are identical. These validation results have been addressed in the results section at line 170, the methods are described in line 649, and the data can be found in supplementary figure 3.

2) vaccination to elicit T cells is reported as a single experiment of 5 mice plus 5 controls. There is variability of course, between these 5 mice. And yeah, it's 5 (five). No repeats. One can argue it's a proof of concept as the authors have done, but the counterargument is that it is a one-off. This is not robust. It may also not be reproducible. There is a reason we repeat experiments in science. This is really not negotiable.

Your comments are appreciated. The mouse *in vivo* experiment has been repeated and the results are added to figure 5, 6, 7 and supplementary figure 8 in the manuscript. The total number of mice has now increased to 10 per group (i.e. two independent experiments of 5 mice/group). This repeat immunogenicity experiment confirmed the results of the first immunogenicity experiment. Additionally, we have improved our analysis of the polyfunctional CD8+ T cells by applying a Boolean gating strategy, providing more information on how many different cytokines the CD8+ T cells are producing.

3) The point of doing this work as a mouse expt is literally to be able to do a challenge. But, no challenge experiment was performed except ex vivo (and with middling results). Without that, the far better experiment is to do this in human (given that the disease we care about is a human disease but also, crucially, given that the epitopes are unlikely to be the same between mouse and human given HLA differences).

Thank you for your assessment, of which we agree. The primary aims of this first manuscript were to (i) demonstrate the utilization of immunopeptidomics to identify novel vaccine targets for Chagas disease that can induced CD8+ cytotoxic T cells that can target Chagas infected host cells, (ii) illustrate the potential of the mRNA platform to induce antigen-specific CD8+ T cell responses, and (iii) rapidly test new candidate antigens found through immunopeptidome screening. We agree that future work will include *in vivo* challenge models to test the efficacy of Tcj2 as a vaccine candidate, hopefully proving that this candidate vaccine antigen can induce significant protection against *T. cruzi* infection in a mouse model, which will pave the way for human vaccine studies. We agree that the identified *T. cruzi* **peptides** presented by the MHC-I molecules of mice and humans will differ, due to the different MHC-I molecules. However, the aim of this study was to identify which of the many *T. cruzi* **proteins** would get access to the MHC-I presentation pathway of the host cell. This will require that *T. cruzi* antigens are released in sufficient quantities from the intracellular parasite into the cytoplasm of the infected host cell, where it can be processed by the proteasome for subsequent presentation of the derived peptides on MHC-I molecules of the infected host cell. The identified MHC-I presented peptides will therefore differ between humans and mice, but the *T. cruzi* antigen that has access to the MHC class I processing pathway (i.e. Tcj2), will most likely be the same for mice and humans.

Given the timeliness and importance of the topic and the work, potentially, and after editorial consultation, the decision is to allow a second major revision. But this would have to incorporate the changes above (at least the 1st and 2nd point; the 3rd is not key).

**Part I - Summary**
Please use this section to discuss strengths/weaknesses of study, novelty/significance, general execution and scholarship.

Reviewer #2: I feel that the authors have done a reasonable job trying to satisfy the majority of the reviewer comments. It is certainly notable that they have elected not to pursue the majority of the experimental suggestions, although I personally feel that the manuscript is robust enough and they have certainly addressed my review comments thoughtfully and completely.

Thank you for your positive comments.

Reviewer #3: The strengths and weaknesses of this study have not significantly changed in this revision. Strengths include generally well executed tackling of vaccine antigen discovery for Chagas disease, a neglected disease and worthy target of such work. The weaknesses remain the conduct of the vaccine antigen discovery work using non-human cells (though this is now much better acknowledged in the revised manuscript), and the very low animal numbers in the single in vivo conducted experiment.

Thank you for your feedback. We agree that there is tremendous value in using human cell lines for T. cruzi research. However, we opted to focus on a murine model system because many of the biological questions about how T. cruzi infects host cells, modulates immune responses, and causes pathology, can be understood by using other mammalian cell lines including murine cells. Future experiments will involve testing different tissues of T. cruzi in infected mice, as well as validation in human cell lines.

**Part II – Major Issues: Key Experiments Required for Acceptance**
Please use this section to detail the key new experiments or modifications of existing experiments that should be absolutely required to validate study conclusions.

Generally, there should be no more than 3 such required experiments or major modifications for a "Major Revision" recommendation. If more than 3 experiments are necessary to validate the study conclusions, then you are encouraged to recommend "Reject".

Reviewer #2: (No Response)

Reviewer #3: (No Response)

**Part III – Minor Issues: Editorial and Data Presentation Modifications**
Please use this section for editorial suggestions as well as relatively minor modifications of existing data that would enhance clarity.

Reviewer #2: (No Response)

Reviewer #3: The main remaining issue is the low mouse numbers in the single in vivo study. As the authors acknowledge, there is presumably funding or other limitations preventing them from replicating the singular small mouse immunogenicity study presented. The work is valid as-is and worthy of publication in this reviewer's judgment - I suspect that the data would repeat well - but this study remains significantly underpowered and without replication. This reviewer leaves it in the editor's ultimate hands as to whether a single study with n=5 mice is sufficient scientific rigor for this particular journal.

Thank you for your feedback. We have repeated the animal immunogenicity study to show reproducibility. The total number of mice has now increased to 10 per group (i.e. two independent experiments of 5 mice/group). The data has been added to figures 5, 6, 7 and supplementary figure 8 in the manuscript. Also, we changed the gating strategy to analyze polyfunctional CD8+ T cells in Figure 6B to a Boolean gating strategy, providing more insights on the different cytokines these CD8+ T cells are secreting.

Other minor issues:

Line 408-409 of the revised manuscript now contain a sentence fragment.

Thank you for noticing this sentence fragment, this has been corrected.

Additional statistical information is appreciated. The use of nonparametric tests is still not necessary, but is not invalid and thus acceptable. The multiple testing corrections mentioned by another reviewer are unnecessary as only two groups are being tested in any one test.

We appreciate your feedback. Throughout the results in figures 5, 6 and 7 we performed a non-parametric statistical test to measure significance using a Mann-Whitney U test. This is mentioned in the methods under the header “data analysis”.

The addition of further information about the mRNA used (i.e., what the UTRs are derived from) is appreciated - and should be added to the Methods section of the paper.

Thank you for your suggestion. The mRNA UTRs are from human β-globin. The complete sequence was codon optimized by the RNAcore at the Houston Methodist Research Institute using their proprietary algorithm which considers dozens of key factors of gene transcription and translation. The codon optimizated mRNA sequence, as well as the plasmid sequence used for in vitro transcription of the mRNA, are property of the RNAcore. We have provided all the available information on the mRNA construct in the methods section of the manuscript.
